# Semiconductor CdF_2_:Ga and CdF_2_:In Crystals as Media for Real-Time Holography

**DOI:** 10.3390/ma5050784

**Published:** 2012-05-07

**Authors:** Alexander I. Ryskin, Alexander S. Shcheulin, Alexander E. Angervaks

**Affiliations:** National Research University of Information Technologies, Mechanics and Optics, 49, Kronverkskiy pr., St. Petersburg 197101, Russia; E-Mails: alex_ryskin@mail.ru (A.I.R.); Shcheulin@oi.ifmo.ru (A.S.S.)

**Keywords:** cadmium fluoride, bistable centers, photoinduced metastable state, holographic grating decay, real-time holography, dynamic correction of wavefronts and optical images, holographic correlator

## Abstract

Monocrystalline cadmium fluoride is a dielectric solid that can be converted into a semiconductor by doping with donor impurities and subsequent heating in the reduction atmosphere. For two donor elements, Ga and In, the donor (“shallow”) state is a metastable one separated from the ground (“deep”) state by a barrier. Photoinduced deep-to-shallow state transition underlies the photochromism of CdF_2_:Ga and CdF_2_:In. Real-time phase holograms are recorded in these crystals capable of following up optical processes in a wide frequency range. The features of photochromic transformations in CdF_2_:Ga and CdF_2_:In crystals as well as holographic characteristics of these media are discussed. Exemplary applications of CdF_2_-based holographic elements are given.

## 1. Introduction

A change in the optical properties of a crystal caused by a change of defect configuration in the lattice allows the crystal to be used as a holographic medium. In particular, such defects are DX-centers in III–V and II–VI semiconductors. This impurity center has a ground state with two valence electrons localized at the atom-like orbital of the impurity and excited hydrogenic (donor) state. Photoinduced change of center state is accompanied by a drastic center reconstruction. This reconstruction creates a barrier separating two states of the center and makes the excited state a metastable one. Features of DX-centers underlie photochromy of semiconductor crystals containing these impurities.

In 1994, the recording of holographic phase grating in an AlGaAs:Si layer created by the photoionization of the ground state of DX-center was demonstrated [1]. The resulting metastable conducting state persisted at sufficiently low temperature, *T*, for an immeasurably long time. Localized modification of free-carrier concentration produced the observed refractive index grating. Similar gratings were also observed in GaAlAs:Te and CdZnTe:Cl [2]. Also, absorption gratings in AlSb:Se were recorded using a bistable defect which did not change free-carrier levels [3]. In 1995, the phase grating was recorded in a wide-gap (7.6 eV) ionic semiconductor, CdF_2_:In, for which variation of the refractive index was produced by photoinduced depopulation of the ground state of In center and the resulting population of its metastable state [4]. Similar grating was recorded in CdF_2_:Ga [5]. As in compound semiconductors, persistent hologram recording is possible in CdF_2_ at a sufficiently low temperature due to a barrier between the metastable and ground states of the impurity center. This barrier determines the time interval for which the grating persists. The temperature increase results in grating decay. Using temperature as a managing parameter ensures realization of the wide range of decay times, *i.e.*, wide range of frequencies of optical processes that can be followed up with these holographic media. Besides, these media have a number of valuable properties, including high spatial resolution of holographic gratings, laser radiation tolerance (unlimited number of recording/readout cycles), and optical isotropy. Large, high-quality CdF_2_ crystals, whose properties make them promising media for the creation of volume elements of real-time holography, are available.

In this review, we consider the mechanism of photoinduced transformation of bistable center state in semiconductor CdF_2_ crystals and the use of crystals with these centers for recording holograms in the real time scale. We argue that these centers are identical to DX-centers in conventional III–V and II–VI semiconductors and point out which features of Ga and In determine their bistable nature in CdF_2_ crystals. The natures of bistable center transformations are discussed in Section 2. Section 3 deals with CdF_2_:In and CdF_2_:Ga crystals as holographic media. In Section 4, some examples of holographic element applications based on these crystals are given.

## 2. Bistable Centers in CdF_2_ Crystals and Their Transformations

### 2.1. The Nature of Bistable Centers in CdF_2_ Crystals

The cadmium fluoride crystal has a fluorite structure (the space symmetry $O_{h}^{5}\left(Fm3m\right)$) that may be presented as a sequence of anion (fluorine) cubes, half of the central positions of which are occupied with cations (cadmium) and the other half are empty, forming interstices. CdF_2_ is an ionic dielectric crystal that can be converted into a semiconducting state via doping with column-III elements of the periodic table (donors). These donors are introduced in the raw material for crystal growth. During subsequent annealing of as-grown crystals in a reducing atmosphere of Cd vapor or in hydrogen (an “additive coloration” of the crystal [6,7]), interstitial fluorine ions, F^−^, which are charge compensators for the excess “+1” charge of the dopants, diffuse out of the volume of the crystal to its surface, where they recombine with the reducing agents (Cd^2+^ or H^+^ ions). The charge neutrality of the crystal is maintained by an opposite current of electrons that are segregated off this agent and diffuse into the crystal volume. These electrons are localized in the conduction band or at hydrogenic donor orbitals (*e_hydr_*) centered on the trivalent impurity ion, thus converting the crystal into a semiconductor state [8,9].

The additive coloration is carried out in the unsoldered quartz ampoule or in a vacuum-processed set-up at temperature *T* = 350–500 °C (the melting temperature of CdF_2_ is 1050 °C).

**Figure 1:**
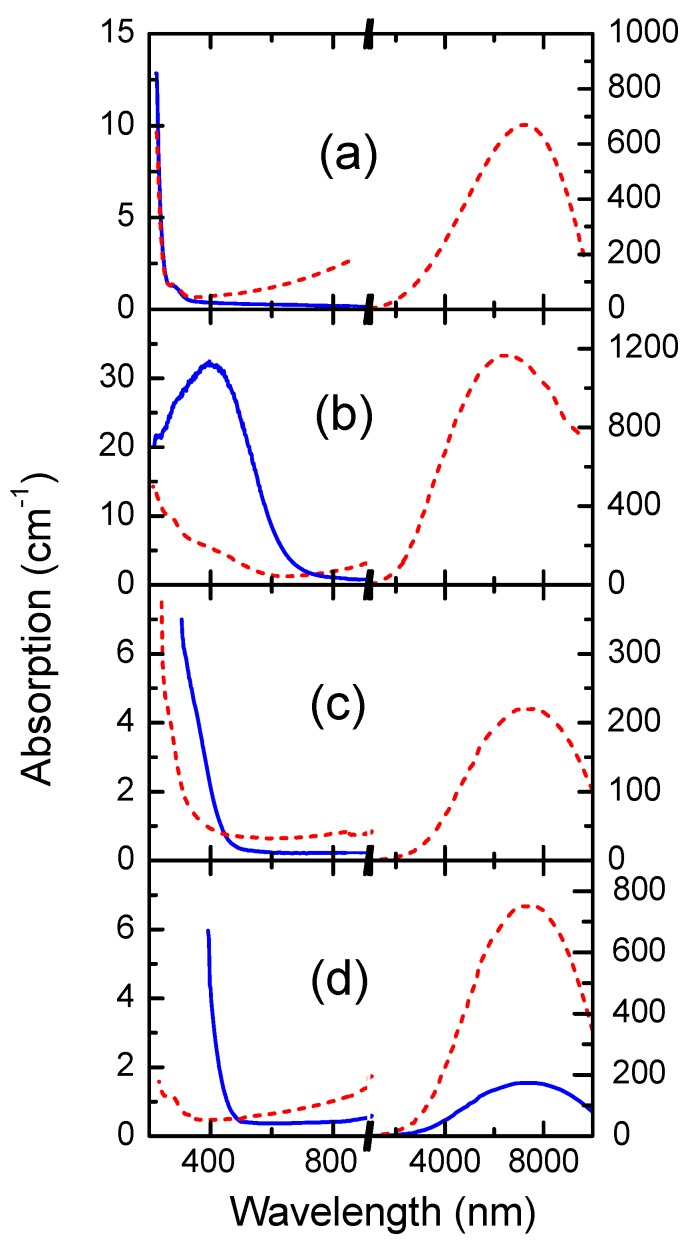
Absorption spectra of (**a**) CdF_2_:Y (*N*_Y_ = 2.2 × 10^18^ cm^−^^3^) at *T* = 77 K before (solid line) and after (dotted line) additive coloration, and spectra of (**b**) CdF_2_:In (*N*_In_ = 4 × 10^18^ cm^−3^); (**c**) CdF_2_:Ga (*N*_Ga_ = 7 × 10^17^ cm^−3^); (**d**) CdF_2_:Ga,Y (*N*_Ga_ = 1.7 × 10^18^ cm^−3^) additively colored crystals cooled in the dark down to *T* = 77 K (solid lines) and the same crystals after subsequent illumination in the UV-VIS absorption band at the same temperature (dotted lines).

The possibility of converting the ionic dielectric crystal into a semiconductor state is a unique feature of CdF_2 _among predominantly ionic crystals. Due to the great electron affinity of CdF_2_, the energy of donor *s*-levels occurs near the bottom of the conduction band and the levels are collectivized with it, forming the hydrogenic orbitals. The binding energy of these orbitals is ~0.1 eV for any donor [10,11]. A wide and intense infrared (IR) absorption band corresponds to the donor photoionization process (Figure 1a; see [11] for calculation of the band’s shape). The band maximum is located at 7–8 µm, depending on the specific impurity.

The constant of interaction of conductivity electrons with longitudinal optical phonons *α* is equal to 3.3 [12]. It follows from this value that the mobile carriers in the conduction band and carriers bound at hydrogenic donor orbitals are a free and bound polaron, respectively.

During the additive coloration procedure, not all interstitial F− ions present in as-grown crystal are substituted for electrons. Similarly to conventional *n*-type semiconductors in which acceptors compensate donors, thus reducing the effective concentration of electrons, ${\tilde{n}}_{\Sigma }$, one may define for CdF_2_ the compensation degree, *K*, by the equation

(1) $${\tilde{n}}_{\Sigma }=N_{\Sigma }(1-K)$$

where *N*_Σ_ is the total donor concentration at sufficiently low temperature (all electrons introduced at additive coloration are localized on donors), whereas *N*_Σ_*K* is the concentration of interstitial F^−^ ions that remain in the crystal after the additive coloration procedure. Probably, this compensation is of non-local nature.

Two donor impurities, Ga and In, form bistable centers in semiconductor CdF_2_ crystals. Like DX-centers, they have the ground and the metastable excited states.

It was initially assumed that, unlike DX-centers, the two states of these impurities in predominantly ionic CdF_2_ crystal correspond to electron localization either at the intrinsic atomic-like orbital (*Me*^2+^ valent state of the impurity, *Me* = Ga, In; “deep” state) or at the hydrogenic orbital (*Me*^3+^ + *e*_hydr_; “shallow” donor state) [13,14,15,16]. Thus, transformation of the center state was assumed to proceed without change in its charge. Within this framework, Ga and In bistable centers were treated as examples of intrinsic self-trapping after Toyozawa (see, for instance, [17]).

Subsequent studies of optical and thermal transformations of *Me* centers in CdF_2_ showed that, in fact, a change in a center’s state is accompanied by a change in the charge. The bimolecular kinetics of thermal destruction of non-equilibrium shallow centers [18] and the quantum yield *η* = 2 of the photoinduced reaction of deep-to-shallow center conversion clearly indicate that two shallow centers participate in the formation of one deep center and *vice versa* [19]. This means that the ground state of the *Me* center corresponds to localization of *two* electrons at the intrinsic atomic-like orbital of the center, thus testifying to the formally single-valent nature of Ga and In ions in the deep state. Accordingly, the photoinduced deep-to-shallow center conversion can be described by Reaction (2):

(2) $$Me^{1+}+Me^{3+}\overset{h\nu }{\to }2\left(Me^{3+}+e_{hydr}\right)$$

As seen from Equation (2), an “empty” *Me*^3+^ ion is an indispensable element of the conversion process.

Direct evidence for the two-electron nature of the deep center was found in measurements of the magnetic moment, *J*, of CdF_2_:In crystals. In these experiments, no magnetic moment was observed in the deep state of the In center. However, it appeared (*J* = ½) when the shallow state occurred, to be populated at photoexcitation of the deep state [20,21].

**Figure 2:**
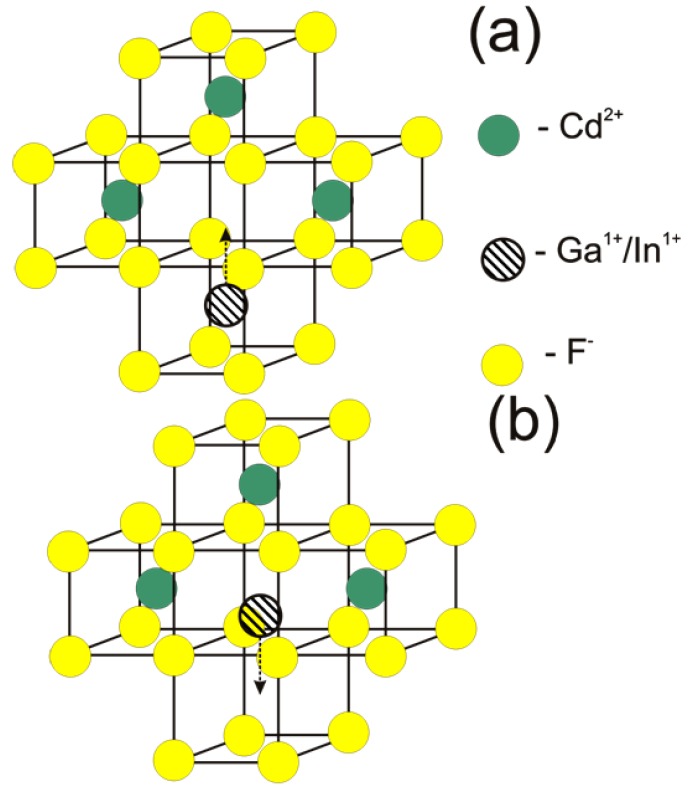
Displacement of a bistable impurity ion at (**a**) formation of a deep center; and (**b**) conversion of a photoionized deep center into a shallow center.

The above facts prove that Ga and In centers in ionic semiconductor CdF_2_ are identical to DX-centers in III–V and II–VI semiconductors. As well as for these centers, the barrier separating the deep and shallow states of the Ga and In centers is due to the center reconstruction when its charge is changed. The microscopic nature of this reconstruction was identified by the first-principle calculation [22]. Formation of the deep state was found to be accompanied by displacement of an impurity from the site position (surrounded by the cube of F^−^ ions) into an adjacent empty cube of anions for a distance of about ¾ of the cube edge (Figure 2a). The presence of vacancy in the deep center structure (after displacement of an impurity) was supported by experiments on positron annihilation in CdF_2_:Ga and CdF_2_:In [23]. The binding energy of the deep state was found to be 0.70 eV for Ga and 0.25 eV for In [22].

A peculiarity of Ga and In, which belong to the boron sub-column of column III of the periodic table, is that for these ions the filled electron shell exists not only for the trivalent *Me*^3+^ state, which is typical for all column-III elements, but also for the univalent *Me*^1+^ state. This state, with two electrons localized with opposite spins on the *ns*-orbital of the impurity (*n* = 4, 5 for Ga and In, respectively), corresponds to the ground state of the center.

Prior to Ga, elements of this sub-column, Al and B, are too small to be incorporated in the site position of the CdF_2_ lattice. T1, which is next after In in the sub-column, was never used as a dopant of this crystal. Elements of the scandium sub-column of column III, in particular rare-earth elements, form only shallow donor states in CdF_2_. Single-valent states of these elements contain one electron in an *ns*-shell and are unstable.

**Figure 3:**
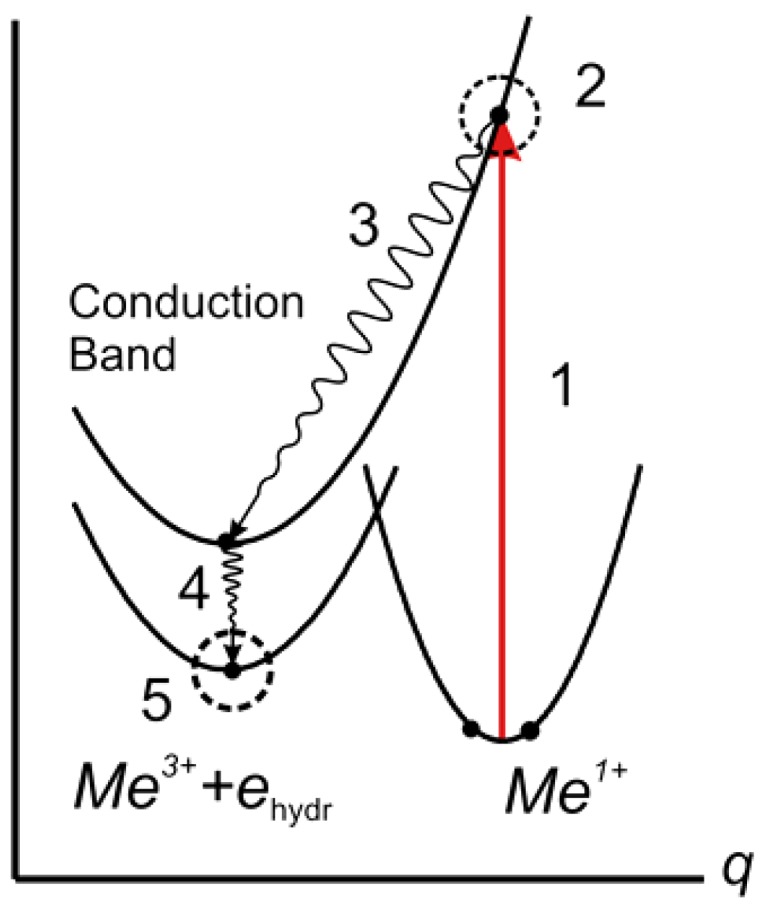
Generalized diagram of configuration coordinates for the states of bistable center in CdF_2_ crystal. Schematic representation of processes 1–5 at deep-to-shallow center transformation are shown (see below in the text). Lattice relaxations that correspond to other configuration coordinates than q, are indicated by dotted circles. Non-radiation transitions with photon emission are shown by wavy lines.

The large lattice relaxation accompanying the deep state formation creates a barrier that separates the shallow donor state from the deep state and makes the shallow state a metastable one (Figure 3). The main feature of this relaxation, *i.e.*, the configuration coordinate of the center, is shown in Figure 2a. The large lattice relaxation is typical for DX-centers and is a consequence of the linear electron-lattice coupling.

**Figure 4:**
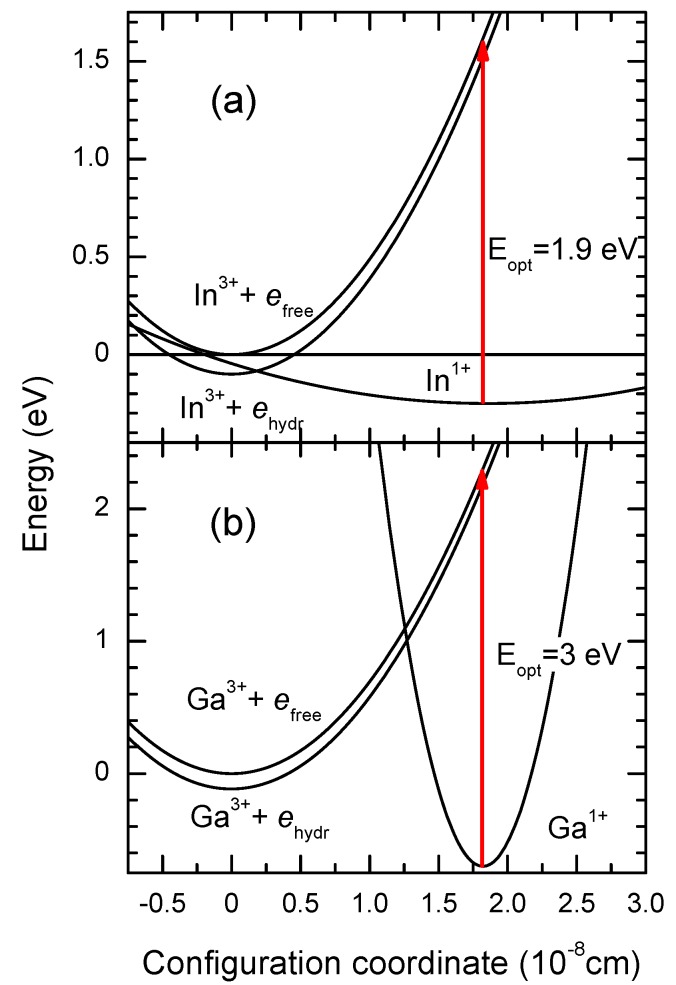
Diagram of configuration coordinates for (**a**) CdF_2_:In; and (**b**) CdF_2_:Ga. The configuration coordinate is the displacement of the impurity ion along the fourth-order axis as it is shown in Figure 2a. *e*_free_ is a free carrier in the conduction band.

Figure 4 shows configuration coordinate diagrams for both ions as calculated in [24] using deep center energies found in [22]. Note the large difference in the barrier height for Ga and In. The main reason for the large barrier in CdF_2_:Ga is the lattice contraction around the relatively small Ga ion [25] that results in more elastic coupling of the impurity with neighboring ions as compared to In.

In absorption spectra of CdF_2_:In and CdF_2_:Ga crystals, the band in the ultraviolet-visible (UV-VIS) spectral range exists, as well as the IR band (Figure 1(b,c); the shape of this band in CdF_2_:Ga crystal is clearly seen in the differential spectrum of this crystal, see Figure 13 below). This wide band has a maximum of ~390 nm for CdF_2_:Ga and ~460 nm for CdF_2_:In; for both crystals it covers the whole of the visible spectrum range and extends up to 830–850 nm. This band is due to photoionization of deep centers. Its shape represents the convolution of the vibronic line-shaped function of optical transition from the deep centers to the conduction band with the density of electronic states in this band.

CdF_2_:In and CdF_2_:Ga crystals cooled in the dark are in a semi-isolating state, since electrons introduced in the course of additive coloration are predominantly located in couples at deep centers. Only the UV-VIS band exists in the absorption spectra of the crystals (Figure 1(b,c)). At excitation in this band, deep-to-shallow state conversion occurs in accordance with the reaction (2) accompanied by UV-VIS band disappearance and IR band occurrence (Figure 1) [26]. The photoexcited shallow state persists below ~220 K in CdF_2_:Ga and below ~40 K in CdF_2_:In [27,28]. At higher temperatures, the thermo-destruction of shallow centers and formation of deep centers occur, both types of center being in equilibrium with each other. Thus, deep-to-shallow and shallow-to-deep center conversion under the impact of temperature is a mutual process that is described by reaction (3):

(3) $$Me^{1+}+Me^{3+}\overset{kT}{↔}2\left(Me^{3+}+e_{hydr}\right)$$

**Figure 5:**
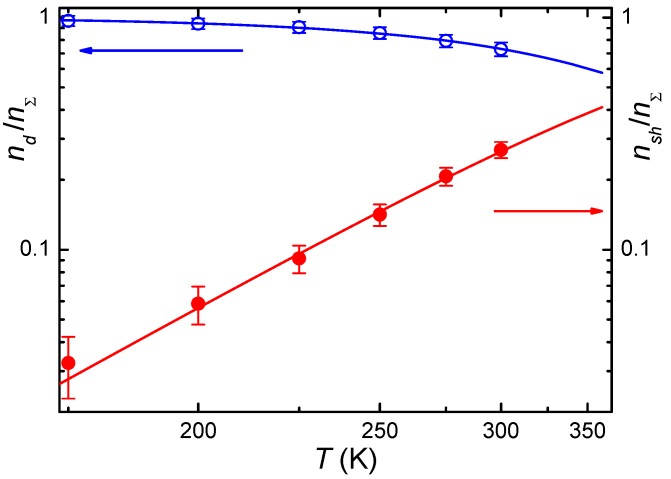
Temperature dependencies of relative concentrations of carriers at deep, n˜dn˜Σ, and shallow, n˜shn˜Σ, centers for CdF_2_:In. Open circles (deep centers) and dark circles (shallow centers) are experimental data; solid lines show theoretical dependency as calculated in [28].

**Figure 6:**
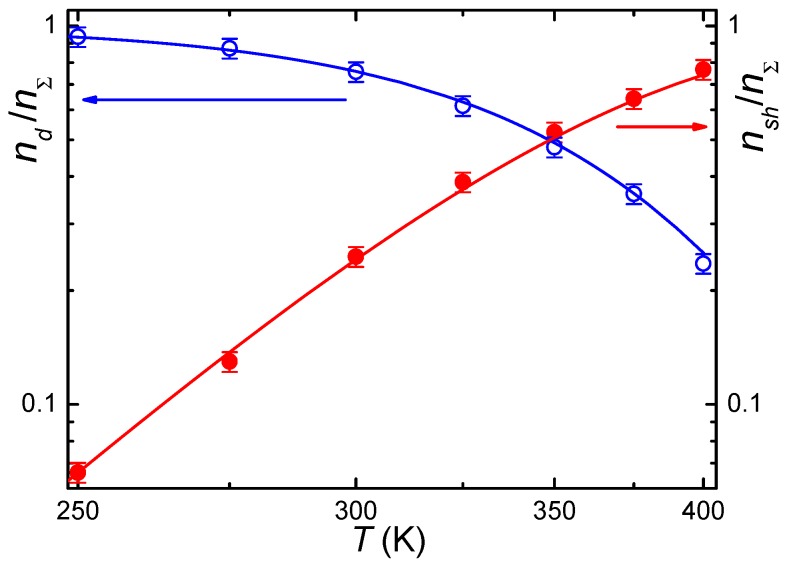
The same as in Figure 5, but for CdF_2_:Ga crystal.

Figures 5 and Figure 6 show the temperature dependencies of relative population of deep and shallow states for both crystals. In principle, electrons introduced in the crystal at its additive coloration are distributed between two states of the impurity and the conduction band. However, for both crystals, the free carrier concentration is very small even at *T* = 400 K [29]. Since the Ga deep state has a higher binding energy as compared with In, and binding energy of the shallow state is nearly equal for both ions, one may propose that the relative population of the shallow state of Ga increases with temperature much more slowly than that of In. Meanwhile, experiment reveals a reverse situation. The difference in the distribution of electrons over levels of two bistable centers was explained in [29] proceeding from statistics of this distribution with allowance for typical dopant compensation degree and relative concentration of impurity centers capable of two-electron deep state formation. It was found that the compensation degree for both dopants is very high: *K* = 0.90–0.97 for CdF_2_:In and *K* = 0.996 for CdF_2_:Ga. This suggests that for both ions the concentration of *Me*^3+^ ions significantly exceeds the maximal concentration of (*Me*^3+^ + *e*_hydr_) centers.

The most important difference between In and Ga dopants in CdF_2_ crystal lies in the number of impurity ions that can form both shallow and deep centers. Whereas for In practically all dopant ions can form these centers, a very small portion of Ga ions present in the crystal (not exceeding one percent) can form the deep centers; meanwhile, most or all of these ions can form the shallow center. This feature of Ga shifts the electron distribution between deep and shallow centers towards the shallow centers and provides faster destruction of Ga deep centers with temperature compared to In. A possible source of this Ga feature is discussed in [29].

Doping CdF_2_ with In, and especially with small-size Ga, increases light scattering in crystals. To reduce this effect, the Ga-doped crystals are co-doped with Y. Co-doping not only decreases the scattering but also diminishes the non-photochromic absorption of these crystals (Figure 1d) [30].

The concentration of centers that can form the deep as well as the shallow state in CdF_2_:Ga crystals (“optically active centers”, which are responsible for the photochromic effect) coincides practically with the concentration of electrons introduced into the crystal during additive coloration, whereas the total Ga concentration in the crystals in accordance with mass-spectrometric data is ~10^20^ cm^−3^. This concentration is determined by the growth conditions and is nearly the same in all Ga-doped crystals.

Co-doping CdF_2_:Ga crystals with Y at the level of 10^19^ cm^−3^ (typical value) increases the concentration of “optically active” Ga ions to approximately twice as many. Co-doping with Y insignificantly changes the temperature dependency of distribution electrons between deep and shallow centers, as shown in Figure 6.

Indium concentration depends on the doping level; it can reach 10^21^ cm^−3^ [31].

The concentration of optically active centers in the specific sample depends on the regime of its additive coloration. As a rule, this concentration does not exceed 10^18^ cm^−3^ for CdF_2_:Ga crystals and 10^19^ cm^−3^ for CdF_2_:In crystals [32].

Shallow donor states in crystals under consideration form the narrow impurity band. For CdF_2_:Ga crystals, the width of this band was estimated as ≤0.2 eV [29] (see also [33]).

Analysis of dependencies shown in Figure 5 and Figure 6 gives lower values of binding energy compared to [23]: this energy was found to be ~0.38 eV for CdF_2_:Ga and ~0.17 eV for CdF_2_:In [29]. These values based on experimental data are probably more trustworthy than values found via the first-principle calculations.

Bearing in mind the following discussion of holographic properties of CdF_2_ crystals with bistable centers one should note such features of semiconductor CdF_2_ crystals as presence of far-IR absorption (10–150 cm^−1^) [34]. It was proposed that this absorption is due to the existence of so-called “ionized donor pairs” in the crystals, near-by disposed couples of donors having one electron (the analog of the molecular hydrogen ion) [35]. It is absent for CdF_2_:Ga and CdF_2_:In crystals cooled in the dark but appears on their illumination in the UV-VIS band.

### 2.2. Kinetics of Photo- and Thermal-Transformations of Bistable Centers

**Figure 7:**
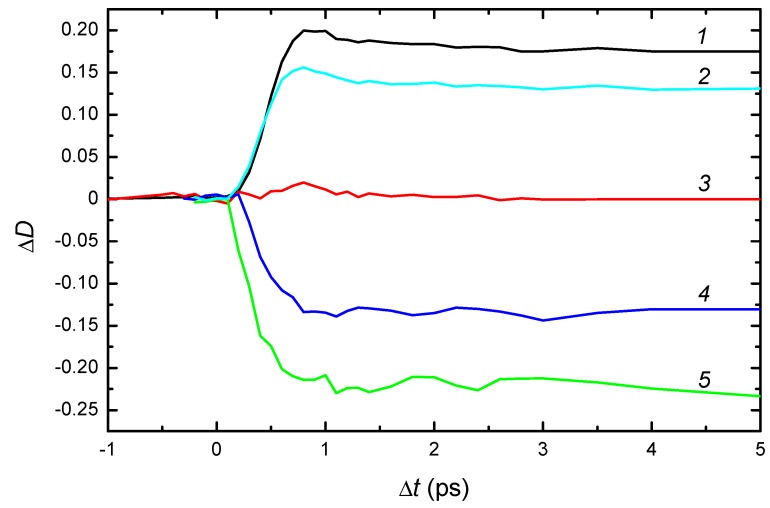
Kinetics of photoinduced time-resolved changes in the absorption spectrum of CdF_2_:In (*N*_In_ = 2.9 × 10^18^ cm^−3^) crystal under the impact of 150-femtosecond laser pulses with wavelength of 395 nm at room temperature. Probe wavelengths are curve *1*: 1100 nm; curve *2*: 1000 nm; curve *3*: 730 nm; curve *4*: 570 nm; and curve *5*: 530 nm. Curve *3* corresponds to minimal absorption in the gap between the UV-VIS and IR bands.

**Figure 8:**
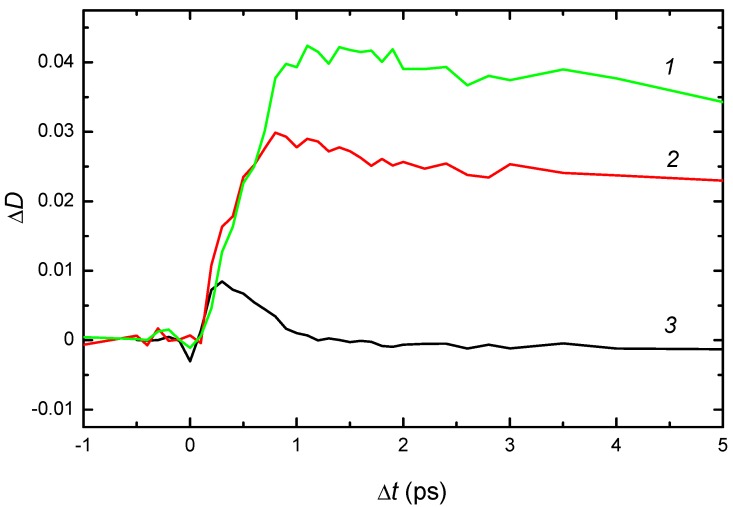
Kinetics of photoinduced time-resolved changes in the absorption spectrum of CdF_2_:Ga (*N*_Ga_ = 4 × 10^17^ cm^−3^) crystal under the impact of 150-femtosecond laser pulses with wavelength of 395 nm at room temperature. Probe wavelengths are curve *1*: 1000 nm; curve *2*: 850 nm and curve *3*: 530 nm.

A time evolution of the photoinduced deep-to-shallow center conversion was studied by recording the absorption spectrum of the crystals at excitation by short UV pulses [36]. Under the impact of 150-femtosecond laser pulses with wavelength of 395 nm, a decrease of absorption in the band of deep centers and an increase of absorption in the band of shallow centers occurs, as recorded by test pulses. The time-resolved changes in absorption of CdF_2_:In and CdF_2_:Ga crystals at several wavelengths in the spectral range of 430–1100 nm are shown in Figure 7 and Figure 8, respectively.

The curve *3* in Figure 7 and Figure 8 are of special interest in terms of the holographic properties of these crystals. These curves confirm the existence of a so-called “isobestic point”, the wavelength for which the increase in the shallow center absorption upon illumination in the UV-VIS band is practically equal to the decrease in the deep center absorption [37]. Actually, this balance is realized not only for a fixed wavelength but for a spectral range in which the absorption bands of both centers overlap (an “isobestic gap”). Within this gap, photoinduced deep-to-shallow state transformation practically does not change the absorption of the crystal.

Mutual compensation of absorption of two centers within this gap takes place not only under photoexcitation of the crystal but even at the change of the crystal temperature.

Curves *4* and *5* in Figure 7 show that the photoinduced transmission band of deep centers is completely formed in a time ~1 ps. This time significantly exceeds both the physical photoionization time of the centers (units of femtoseconds) and the time-resolution function of the spectrometer (~400 fs). This means that the deep center photoionization does not complete the formation of the transmission band of deep centers, which reflects the deep-to-shallow center conversion. The point is that the photoionization time is too short for a noticeable change in the space configuration of the ionized deep center. The one-electron state of such a center is unstable because its nuclear configuration corresponds to the deep (two-electron) state. This instability leads to rearrangement of the lattice, shown in Figure 3b. The rearrangement occurs in a time equal to reverse vibrational frequencies (10^−13^–10^−12^ s). This process determines the dynamics of UV-VIS transmission band formation.

The nature and origin of transient processes in the photoinduced IR absorption (curves *1* and *2* in Figure 7 and Figure 8) are more complex. The IR response is formed by a number of processes, including the following: (1) photo-detachment of an electron from a deep center to the conduction band; (2) subsequent transformation of this electron into a free polaron; (3) polaron relaxation to the bottom of the conduction band; (4) capture of the polaron by an *Me*^3+^ ion; (5) transformation of this complex into the hydrogen-like shallow center (the bound polaron); (6) formation of a similar center as a result of the transformation of an ionized deep center; the corresponding lattice relaxation is shown in Figure 3b; and (7) establishment of temperature equilibrium in the system of bistable centers in the deep and shallow states and free polarons. Processes 1–5 are shown schematically in Figure 3.

The important circumstance for interpreting the photoinduced IR absorption is that the free-carrier absorption cross-section in the near-IR spectral range exceeds the cross-section of shallow centers by a factor of three to four [38]. Hence, the initial stage of the rise of optical density with time for the IR band up to the maximum is mainly due to absorption by photoinduced free carriers (electrons and polarons). A kink (shoulder) on the increasing edge of IR response (curves *1* and *2* in Figure 8) probably divides stages corresponding to free-electron and free-polaron absorption. This feature allows estimation of the time of polaron formation in CdF_2_ as 0.8–1.2 ps; this time is similar to that of lattice rearrangement at the conversion of the ionized deep center into the shallow one. Its presence in CdF_2_:Ga and absence in CdF_2_:In crystals is explained in [36].

The relative contribution of the shallow-center absorption increases as free polarons are captured by *Me*^3+^ ions. The time of this capturing is comparable with polaron formation and lattice rearrangement times due to a large concentration of *Me*^3+^ ions (the high compensation degree).

The temperature-dependent decay of photoinduced (non-equilibrium) shallow centers is a much slower process compared with the process of formation of these centers. According to reaction (3), it obeys bimolecular kinetics, for which shallow center concentration is low compared to the concentration of *Me*^3+^ ions. The above-mentioned difference between In and Ga dopants (see Section 2.1) results in different equations describing the shallow center decay for these two impurities (Equations (4) and (5), respectively [39]:

(4) $$\frac{d\tilde{n}}{C(T)dt}=-{\tilde{n}}^{2}+A(T)(1-\tilde{n})$$

(5) $$\frac{d\tilde{n}}{C(T)dt}=-{\tilde{n}}^{2}(1+\tilde{n})+A(T)(1-\tilde{n})$$

Here n˜=n˜shn˜Σ is the relative concentration of shallow centers, *C*(*T*) is the temperature-dependent parameter related to the shallow center decay, and *A*(*T*) is the parameter that characterizes the equilibrium concentration of these centers. The solution of Equation (4) is of the hyperbolic-cotangent type and it converts into an exponent at the final stages of decay. For Equation (5), only a numerical solution is possible. As well as in Equation (4), its final stages are exponential ones. As temperature increases, the exponential stage embraces the growing part of the decay curve. At sufficiently high temperature, the decay is practically a purely exponential one.

The *C*(*T*) parameter in Equations (4) and (5) determines the rate of formation of deep centers from the shallow centers. The rate of the inverse process is determined by the parameter *B*(*T*) = *A*(*T*)*C*(*T*). The dimensionless *A*(*T*) parameter is determined by the relative concentration of shallow centers in thermal equilibrium conditions, ${\tilde{n}}^{0}(T)=\frac{{\tilde{n}}_{sh}^{0}}{{\tilde{n}}_{\Sigma }}$, where ${\tilde{n}}^{0}s_{h}$ is an equilibrium concentration of these centers:

(6) $$A(T)=\frac{{\left[{\tilde{n}}^{0}\right]}^{2}}{1-{\tilde{n}}^{0}(T)}$$

*A*(*T*) dependency is calculated from the temperature dependencies of equilibrium concentrations (Figure 5 and Figure 6). Then, Equation (4) or Equation (5) is solved for a set of temperatures, and the solutions are compared with experimental decay curves. *C*(*T*) is found as a best-fit parameter. Then, *B*(*T*) dependence is calculated in accordance with the above-given relation. Temperature dependencies of all parameters lie on the Arrhenius plot.

**Figure 9:**
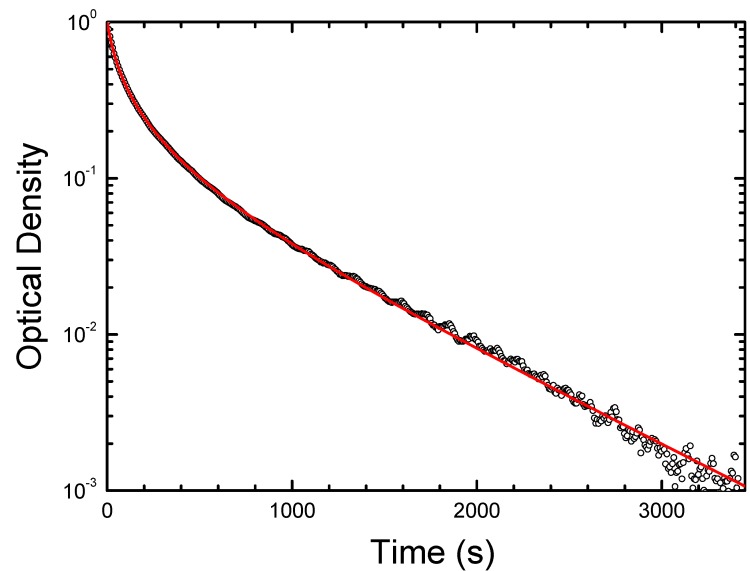
Restoration of the equilibrium concentration of shallow donor states after stoppage of excitation in the UV-VIS absorption band (at *t* = 0) in CdF_2_:Ga (*N*_Ga_ = 4 × 10^17^ cm^−3^) crystal at *T* = 254 K. Points are experimental data; solid line represents the solution of Equation (5) with best-fit parameters.

**Figure 10:**
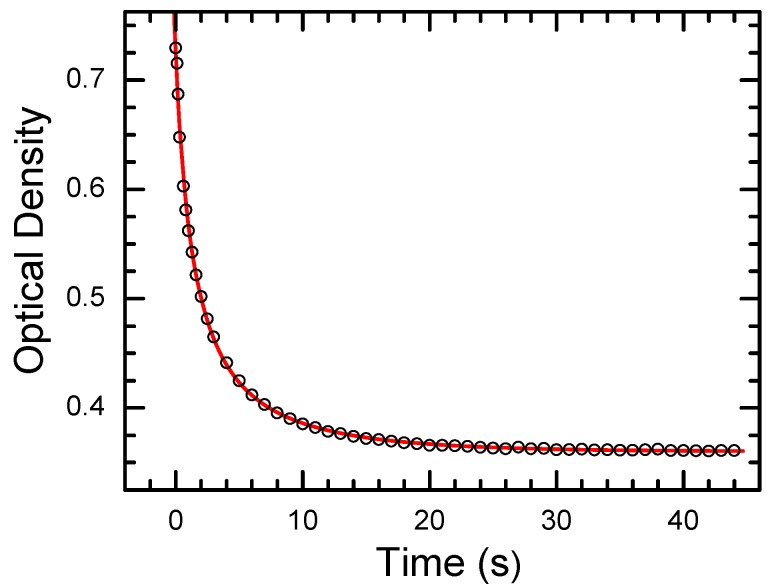
Restoration of the equilibrium concentration of shallow donor states after stoppage of excitation in the UV-VIS absorption band (at *t* = 0) in CdF_2_:Ga (*N*_Ga_ = 4 × 10^17^ cm^−3^) crystal at *T* = 294 K. Points are experimental data; solid line represents an exponent.

As an example, the decay of photoinduced concentration of shallow centers at *T* = 254 K for CdF_2_:Ga crystal and its approximation by Equation (5) are shown in Figure 9. The “high-temperature” (nearly exponential) decay for this crystal is shown in Figure 10.

Using *C*(*T*) and *B*(*T*) dependencies, the barrier height, *E*_bar_, can be found (see [39] for details). For CdF:Ga, *E*_bar_ = 0.630 ± 0.010 eV; for CdF:In, *E*_bar_ = 0.125 ± 0.010 eV.

## 3. CdF_2_ Crystals with Bistable Impurity Centers as a Holographic Media

### 3.1. Diffraction Efficiency and Hologram Decay

As follows from Section 2, the photochromy of CdF_2_ crystals with bistable impurity centers is based on photoinduced conversion of the deep centers into the shallow centers. A hologram recording is possible in the spectral range of the UV-VIS absorption band. When exciting the crystal in this band, electrons tightly bound at the deep centers are substituted for weakly bound electrons located at shallow donor centers or in the conduction band that is in equilibrium with these centers. The spatial modulation of photoinduced shallow centers concentration results in modulation of optical constants of the crystal, *i.e*., formation of holographic grating. Because of the metastable nature of the shallow state, the processes of grating creation (due to deep-to-shallow center conversion, the “direct” process) and decay (due to thermal-induced destruction of non-equilibrium shallow centers, the “reverse” process) progress simultaneously [40].

**Figure 11:**
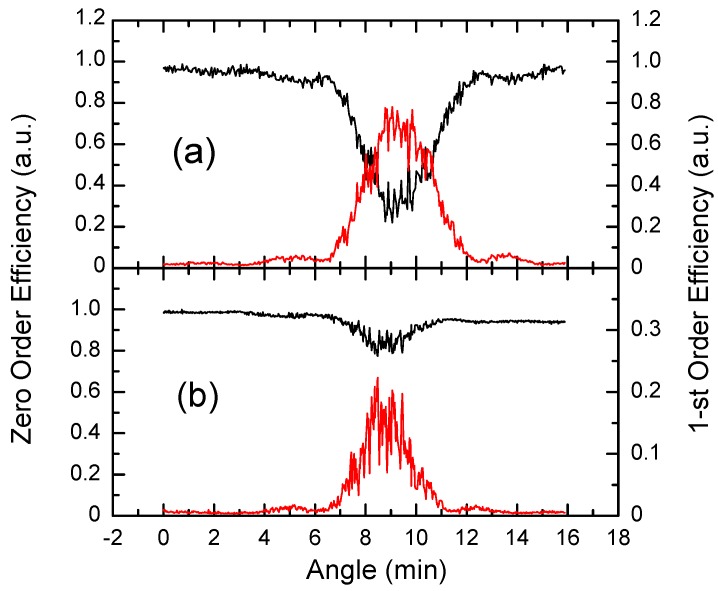
Angular dependencies of the zero-(black lines) and first-order (red lines) diffraction responses of CdF_2_:Ga,Y crystal with hologram at readout by (**a**) 532 nm and (**b**) 660 nm radiation.

The photoinduced change of absorption spectra of CdF_2_ crystals with bistable centers is shown in Figures 1(b–d). The existence of the “isobestic gap”, in which the long-wavelength tail of UV-VIS is superimposed on the short-wavelength tail of the IR band (see Section 2.2) so that deep-to-shallow center conversion does not change an absorption, allows the reading out of predominantly phase holograms. This gap embraces the range of 450–650 nm for CdF_2_:Ga and CdF_2_:Ga,Y and 680–730 nm for CdF_2_:In. Figure 11 shows angular dependencies of the zero- and the first-order diffraction responses for two readout wavelengths in the CdF_2_:Ga,Y crystal isobestic gap. The hologram was recorded by 532 nm radiation in a 10 × 10 × 10 mm^3^ sample and readout by 532 nm and 660 nm radiation. As is seen from Figure 11, the minima in the zero-order response coincide with the maxima of the first-order response that proves the phase nature of the holograms. One can see significant signal oscillations in Figure 11. Their origin is explained below.

**Figure 12:**
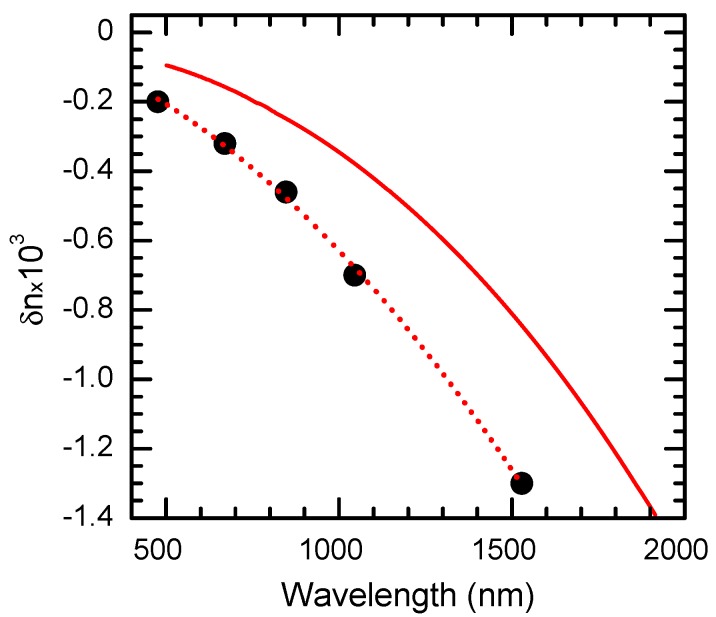
Spectral dependency of the photoinduced change of refractive index of CdF_2_:Ga (*N*_Ga_ = 4 × 10^17^ cm^−3^) crystal determined at *T* = 100 K (black circles) and its fit with the two-oscillator model (dotted line). The solid line shows this dependence calculated by the Kramers-Kronig transformation using the photoinduced modification of absorption spectrum of the crystal that is shown in Figure 13.

Figure 12 shows the spectral dependency of *δn* for CdF_2_:Ga crystal. The measurements in the spectral range of 457–837 nm were executed by means of a Mach-Zehnder interferometer [41].

This dependency can be fit by a two-oscillator model that includes high- (*ω*_1_) and low-frequency (*ω*_2_) effective oscillators [4,41]:

(7) $$\delta n\left(\omega \right)=\frac{2\pi e^{2}}{mn}\left(\frac{\Delta N_{1}f_{1}}{\omega_{1}^{2}-\omega^{2}}+\frac{\Delta N_{2}f_{2}}{\omega_{2}^{2}-\omega^{2}}\right)$$

where *e* and* m* are the electron charge and mass, respectively, Δ*N*_i_ is the photoinduced change of the *i*-th oscillator concentration, and *f*_i _is the *i*-th oscillator strength (*i* = 1, 2). Let us suppose that for frequencies in the spectral range for which *δn* is determined, the following inequality is satisfied: *ω*_1_ >>* ω* >>* ω*_2_. Then, after neglecting *ω* and *ω*_2_ in the denominator of the first and second terms of Equation (7), respectively, and substituting *ω* for 2π*c*/*λ* (*c* is the light speed in vacuum) in the second term, Equation (7) can be presented in the form:

(8) $$\delta n\left(\lambda \right)≅a-b\lambda^{2}$$

where $a=2\pi e^{2}\Delta N_{1}f_{1}/mn\omega_{1}^{2}$ and $b=e^{2}\Delta N_{2}f_{2}c/2\pi mn$ are the best-fit parameters of the model. The negative sign of the experimentally determined *δn* testifies that the parameter *a* is negative and *b* is positive, which corresponds to a decrease of “high-frequency” centers (Δ*N*_1_ < 0) and an increase in “low-frequency” centers (Δ*N*_2_ > 0) in the process of holographic grating recording. It is evident from Equation (8) that the high-frequency oscillator is responsible for the constant (spectrally independent) shift in *δn* whereas the low-frequency oscillator determines the quadratic character of the *δn*(*λ*) dependency.

The experimental spectral dependency of *δn* is well described by Equation (8) (Figure 12). Figure 12 shows that the low-frequency oscillator brings the major contribution to *δn* (also see below).

Assuming the total completion of reaction (2) and using the Kramers-Kronig transformation

(9) $$\delta n(\nu_{1})=\int_{0}^{\infty }\frac{\delta \alpha (\nu )\nu d\nu }{\nu^{2}-\nu_{1}^{2}}$$

where *ν*, *ν*_1_ are wave numbers, one can calculate from the change of the absorption coefficient *δα*(*ν*) the spectral dependency of the photoinduced modification of the refractive index of the crystal *δn*(*ν*), which determines the diffraction efficiency of grating recorded in the crystal.

**Figure 13:**
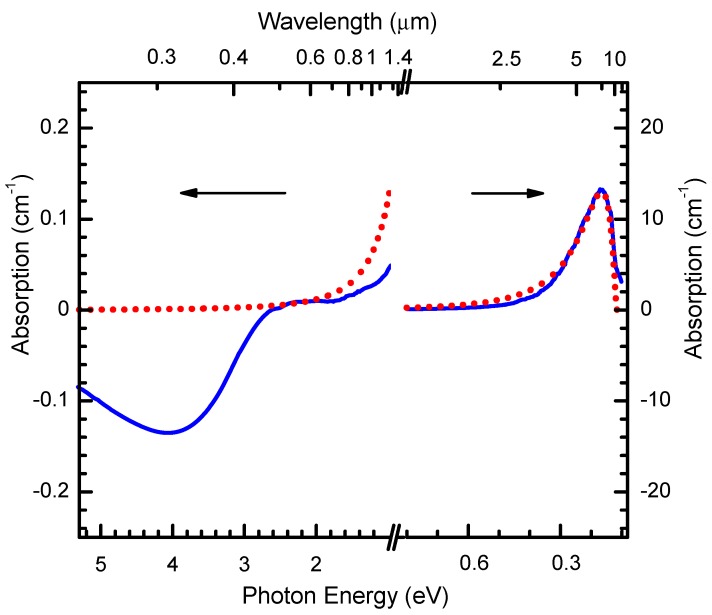
Photoinduced modification of the absorption spectrum of CdF_2_:Ga (*N*_Ga_ = 4 × 10^17^ cm^−3^) crystal cooled in the dark down to *T* = 77 K after illumination in the UV-VIS band at the same temperature (solid line). The dotted line shows an approximation of the IR band using the theory of [11].

The modification of the absorption spectrum (differential spectrum) of CdF_2_:Ga crystal in the photon-energy range of 0.09–5.5 eV at *T* = 77 K is shown in Figure 13. The dependency *δn*(*λ*) calculated from Equation (9) using this spectrum is illustrated by the solid line in Figure 12. The comparison of such dependency with the best-fit spectral dependency (8) shows that the photoinduced IR band is responsible for no more than 70% of refractive index modification. In other words, the contribution of this band to the low-frequency oscillator does not exceed 70%. One can propose that the other 30% is due to ionized donor pair absorption that arises at photoexcitation of deep centers (see Section 2.1).

Another approach to the calculation of the refractive index variation is based on the fact that for probing photon frequencies much higher than frequencies of optical transitions of the weakly bound electrons of hydrogenic centers, the contribution of donor electrons to the refractive index change can be represented by the same expression as for free-electron plasma [42]:

(10) $$\delta n=-\frac{2\pi N_{hydr}e^{2}}{nm*\omega^{2}}=-\frac{N_{hydr}e^{2}\lambda^{2}}{2\pi c^{2}nm*}$$

with *N_hydr_* = 7 × 10^17^ cm^−3^ [41], *n* = 1.575, and polaron mass m* = 0.9, we find, for the readout wavelength of 476 nm, *δn* = −2 × 10^−4^, which is in reasonable agreement with the experimental value. This conclusion states that the change in the shallow center concentration determines the strength of the phase grating that is read out in the isobestic gap.

In accordance with Kogelnik’s theory [43], the diffraction efficiency, *η*, for the phase sinusoidal grating is determined by the formula

(11) $$\eta ={\text{sin}}^{2}\left(\frac{\pi n_{1}d}{\lambda_{0}\text{cos}\theta }\right)$$

where the refractive index is assumed to be spatially modulated in the form *n*(*x*) = *n*_0_ + *n*_1_sin*x*. In Equation (11), *d* is the grating thickness, *λ*_0_ is the readout wavelength in a vacuum, and *θ* is the angle between the readout beam and the normal to the grating surface inside the crystal volume. For not too high values of *η* (practically, for *η* < 0.7), this quantity has quadratic dependency on *δn* or, according to (10), on the concentration of the photoinduced shallow centers.

In accordance with Equations (8) and (11), for *η* < 0.7, *η* = (*a* − *bλ*^2^)^2^. A quadratic dependency of *η* on the exposure at the initial stage of the dependency means that both *a* and *b* coefficients depend linearly on the exposure. This conclusion agrees with the nature of *η* dependency on the recording light intensity [44].

Discussing the dependency of *η* on exposure and on temperature (see below), one should bear in mind that the crystal with DX-center is a reversible medium. The maximum value of the diffraction efficiency at the given power density of radiation and given temperature corresponds to equilibrium of the direct and reverse processes of center transformation.

**Figure 14:**
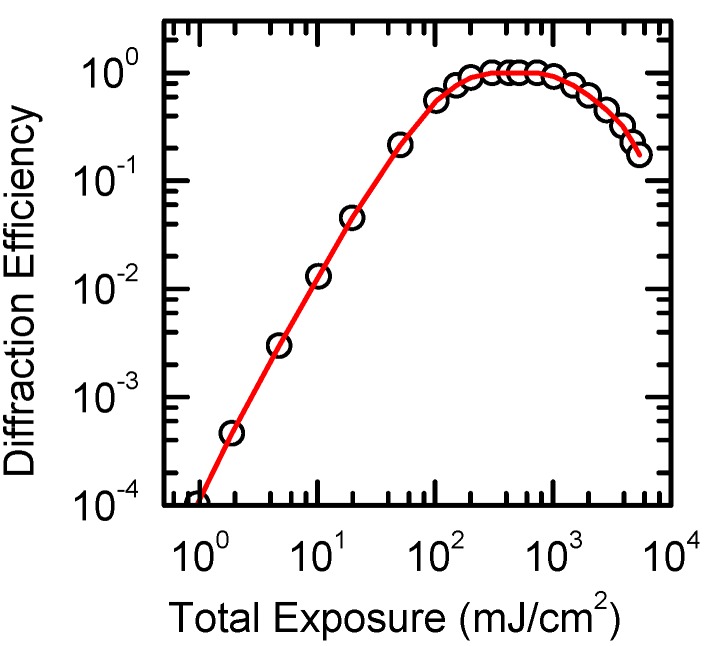
Diffraction efficiency of holographic grating recorded at *T* = 100 K in CdF_2_:Ga (*N*_Ga_ = 4 × 10^17^ cm^−3^) crystal *vs.* exposure. Recording and readout at λ = 453 nm.

Figure 14 shows the dependency of the diffraction efficiency of the grating recorded in CdF_2_:Ga crystal at *T* = 100 K on exposure; the recording and readout of the grating were executed at wavelength *λ* = 453 nm. At the recording temperature of 100 K, the photoinduced shallow centers are persistent (see Section 2.1). It means that the reverse process is “frozen”. The dependency in Figure 14 exhibits typical saturation behavior associated with this type of holographic material. The diffraction efficiency increases with the square of exposure for weak exposures, saturates as the deep centers become depleted and the shape of the grating departs from pure sinusoidal, and diminishes at further exposure growth. The decrease of *η* after attaining the saturation value is due to the deep-to-shallow center conversion in the minima of the fringe pattern because of the light scattering in the sample.

At *T* > 220 K, the reverse process works and diffraction efficiency depends not on the exposition but on the power density of the recording radiation. To get the maximum diffraction efficiency at the given temperature, the power density should be high enough to realize deep-to-shallow center conversion for a time shorter than the decay time of the hologram. As a rule, such power density demands a pulse excitation.

**Figure 15:**
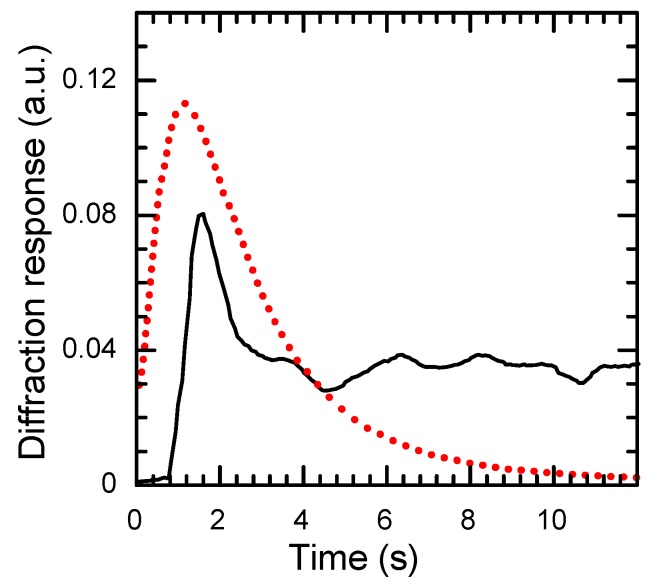
Kinetics of the hologram recording (solid line) and decay (dotted line) in CdF_2_:Ga,Y (*N*_Ga_ = 1.1 × 10^18^ cm^−3^) crystal at room temperature. Recording at λ = 532 nm, readout at λ = 632.8 nm. The maximal diffraction response at both switching and switching-off the recording radiation correspond to the phase incursion of λ/4. The one-second delay at switching the recording radiation is due to the response time of the gate cutting off the radiation.

The reversible nature of this media displays itself in oscillations of the diffraction response at deficient mechanical stability of the optical scheme during the hologram recording process (Figure 15) [40]. At bench vibrations, displacement of the fringe pattern occurs and the hologram recording begins in a new position, while the hologram in the previous position does not erase. As a result, the hologram recorded in a thick sample is a noticeably inhomogeneous one that leads to chaotic change of diffraction response during the recording process. This inhomogeneity also appears in the form of oscillations of the angular dependencies of diffraction response (Figure 11). According to the phase nature of holograms, the zero-order and first-order oscillations at any angular position have equal but oppositely-signed amplitudes.

**Figure 16:**
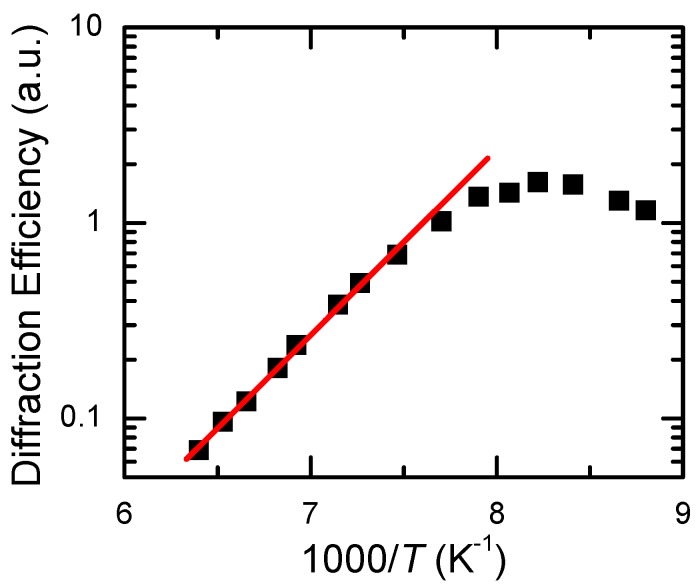
Temperature dependency of diffraction efficiency of the hologram recorded in CdF_2_:In (*N*_In_ = 2 × 10^18^ cm^−3^) crystal by argon laser radiation (*λ* = 514 nm).

The diffraction efficiency of grating recorded on bistable centers in CdF_2_:In crystal reaches a maximum at temperature *T*_max_ that depends on the power density of the recording radiation. Both decrease and increase of *T* relative to *T*_max_ result in decrease of *η* (Figure 16). The “low-temperature” decrease is due to erasing the grating as a result of accumulation of shallow centers in minima of the fringe pattern induced by light scattering in the sample. The “high-temperature” decrease is determined by the increasing role of the reverse process with the temperature growth. A considerable part of this wing lies on the Arrhenius plot; in this part, the decay is nearly exponential (see Section 2.2).

**Figure 17:**
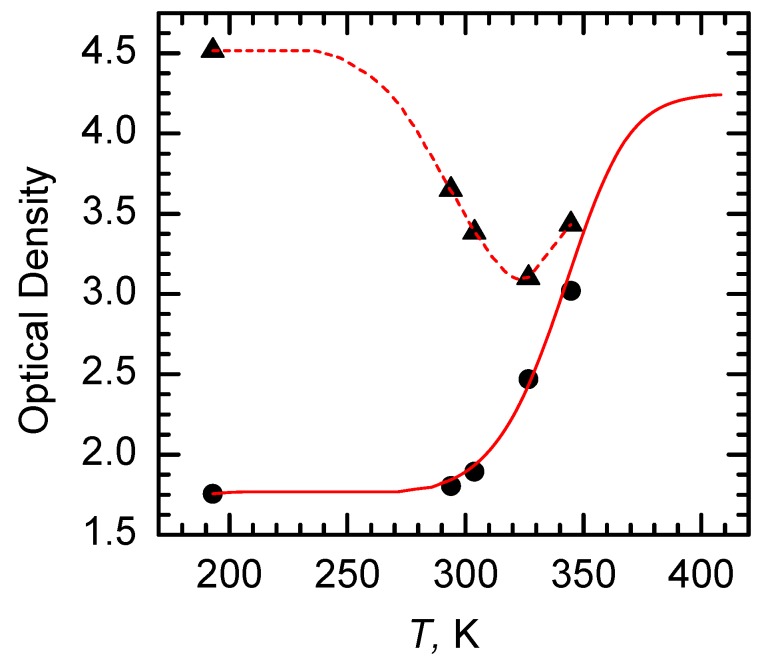
Temperature dependency of IR absorption at *λ* = 1.3 µm in CdF_2_:Ga,Y (*N*_Ga_ = 5.7 × 10^17^ cm^−3^) crystal in the dark (dark circles are experimental points; solid line is the result of calculation based on Figure 6 data) and under excitation of argon laser radiation (dark triangles are experimental points; dashed line shows a trend).

It was stated above that the diffraction efficiency of a hologram recorded in CdF_2_ crystals with bistable centers depends on the photoinduced concentration of shallow centers. Figure 17 shows IR absorption of CdF_2_:Ga,Y crystals in the temperature range of 193–344 K in the dark and after illumination by argon laser (*λ* = 488 nm) up to saturation of the optical density [45]. Absorption was measured at the wavelength of 1.3 µm. The shape of the IR band weakly depends on temperature so the temperature dependency of IR absorption can be measured at the fixed wavelength. The “dark” absorption follows the temperature dependency of shallow center concentration (Figure 6); its increase reflects the thermal population of shallow levels. The light-induced absorption at *T* = 193 K corresponds to the total deep-to-shallow center conversion.

Figure 17 reveals that beginning with *T* ~ 250 K a total deep-to-shallow center conversion becomes impossible (at finite power density of exciting light of 1.5 W/cm^2^) due to thermal destruction of deep centers; this is the origin of the decrease of the photoinduced absorption with increasing *T* in the range of 250–320 K. However, a further increase of *T* results in an increase of this absorption because of the growth of the equilibrium population of the shallow centers. The difference between the upper and lower curves shows the photoinduced absorption; the corresponding refractive index change determines the diffraction efficiency of the hologram.

**Figure 18:**
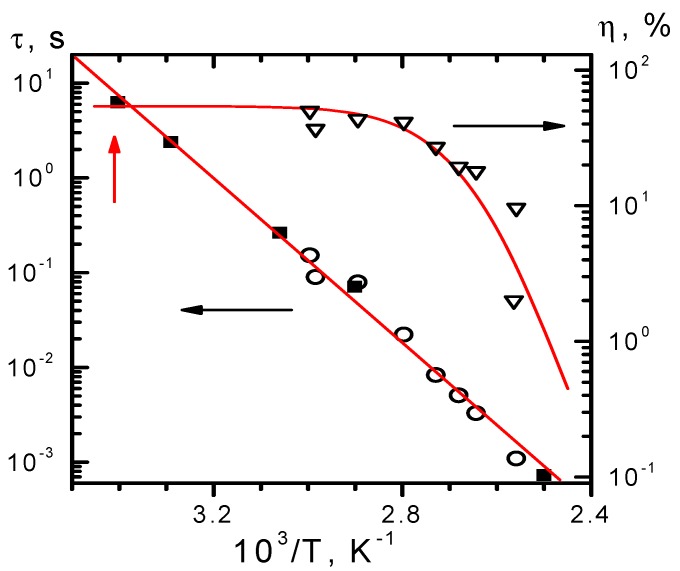
Temperature dependencies of diffraction efficiency (open triangles are experimental data; solid line is the result of calculation based on temperature dependence of the shallow center concentration shown in Figure 6) and the time of ten-fold decay of IR absorption (dark squares) and hundred-fold decay of initial diffraction efficiency (open circles) of the hologram recorded in CdF_2_:Ga,Y (*N*_Ga_ = 5.7 × 10^17^ cm^−3^) crystal by pulses of the second harmonics of Nd:YAG laser. The solid line is an approximation of decay data by an Arrhenius plot. Data that correspond to room temperature are indicated by the vertical arrow.

**Figure 19:**
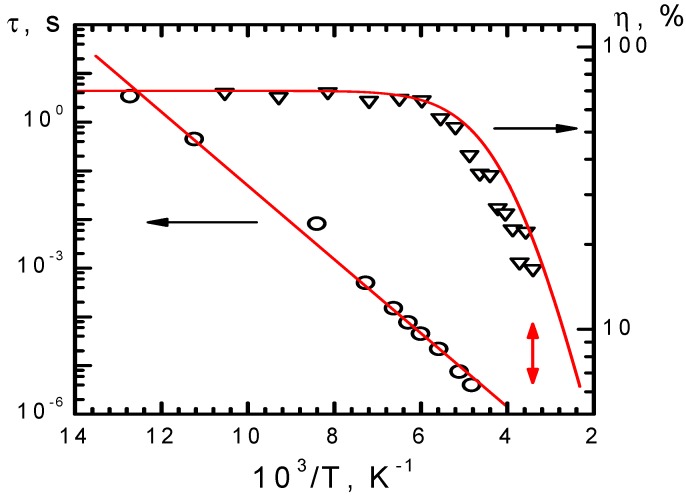
Temperature dependencies of diffraction efficiency (open triangles are experimental data; solid line is the result of calculation based on temperature dependence of the shallow center concentration shown in Figure 5) and the time of hundred-fold decay of initial diffraction efficiency (open circles) of the hologram recorded in CdF_2_:In (*N*_In_ = 4 × 10^18^ cm^−3^) crystal by pulses of the ruby laser. The solid line is an approximation of decay data by an Arrhenius plot. Data that correspond to room temperature are indicated by the vertical arrow.

To characterize the crystal response for real-time holography, the temperature dependencies of diffraction efficiency and decay time of phase holograms were studied for the temperature ranges of 300–400 K (CdF_2_:Ga,Y, Figure 18) and 77–300 K (CdF_2_:In, Figure 19) [45,46,47]. The hologram recording was executed using single pulses of second harmonic of Nd:YAG laser at 532 nm of 20 ns duration with pulse energy equal to 400 mJ/cm^2^ (CdF_2_:Ga,Y), and single pulses of ruby laser at 693 nm of 50 ns duration with pulse energy equal to 300 mJ/cm^2^ (CdF_2_:In). The hologram readout for these crystals was executed using He-Ne laser. For both crystals, the hologram recording and readout was performed within the isobestic gap. The hologram decay down to 1/100 of the initial value of diffraction efficiency was determined using the oscillographic technique. For CdF_2_:Ga,Y, the photoinduced IR absorption decay was recorded together with the hologram decay, taking into account that, in accordance with Equation (11), the hundred-fold decrease of the diffraction efficiency corresponds to a ten-fold decrease of the refractive index (the shallow center concentration).

The crystal thickness was 5 mm for CdF_2_:Ga,Y and 2 mm for CdF_2_:In. These thicknesses ensure, at sufficiently low temperature, the *π*/2 nonlinear phase shift, *i.e.*, the maximal diffraction efficiency of holograms.

The low-temperature diffraction efficiencies of holograms are limited by the transmission of the crystal at the readout wavelength (54% for CdF_2_:Ga,Y and 70% for CdF_2_:In). The temperature dependency of diffraction efficiency is determined by the equilibrium population of shallow centers; the growth of this population with temperature decreases their photoinduced modification. It is seen from Figure 18 and Figure 19 that for both crystals (1) the pulse energy is enough for the total deep-to-shallow center conversion within the pulse and (2) the pulse duration is sufficiently short and does not influence the diffraction efficiency for the studied range of decay times of the hologram.

For both crystals, decay time lies on the Arrhenius plot, indicating that in the temperature range under consideration the hologram decay is practically exponential. Activation energies of decay time dependencies on the temperature are 0.84 eV for CdF_2_:Ga,Y and 0.15 eV for CdF_2_:In.

Outside the isobestic gap, holograms are of amplitude-phase nature. Allowing for the fact that *δn* is negative and *δα* is negative for the UV-VIS absorption band and positive for the IR band, one may conclude that the grating in the spectral range abutted on the short-wavelength border of the isobestic gap has *π*-out-of-phase character, whereas the grating in the spectral range abutted on the long-wavelength border of this gap has in-phase character.

Hologram readouts in the spectral range of the IR absorption band (~0.8–12 µm) could be of special interest. Due to quadratic dependency of the photoinduced refractive index on the readout wavelength, *δn* may vary with *λ* increase in this range from units of 10^−4^ up to units of 10^−2^, *i.e.*, it has a relatively large value. In spite of the large optical density of the IR band (see Figure 1), the transmission at the readout wavelength can be sufficiently large due to the Borrmann effect. In fact, if the equilibrium population of shallow centers is small the total deep-to-shallow center conversion creates high contrast of the diffraction fringe patterns, which favors this effect, *i.e.*, ensures relatively high crystal transmission at the Bragg angle. Therefore, the forming of effective amplitude-phase holograms is possible in the IR spectral range. The diffraction efficiency of a hologram should be maximal at low temperature. It should diminish with increasing temperature due to both a decrease in photoinduced modulation of optical constants and deterioration of conditions for the Borrmann effect display.

One should note that, unlike UV-VIS, IR radiation does not provoke the photochromic effect, *i.e.*, it does not cancel the hologram [48]. However, the decrease of diffraction efficiency of the hologram can be accounted for by heating of the crystal by readout radiation [44].

### 3.2. Holographic Media Resolution, Sensitivity and Operation Mode

**Figure 20:**
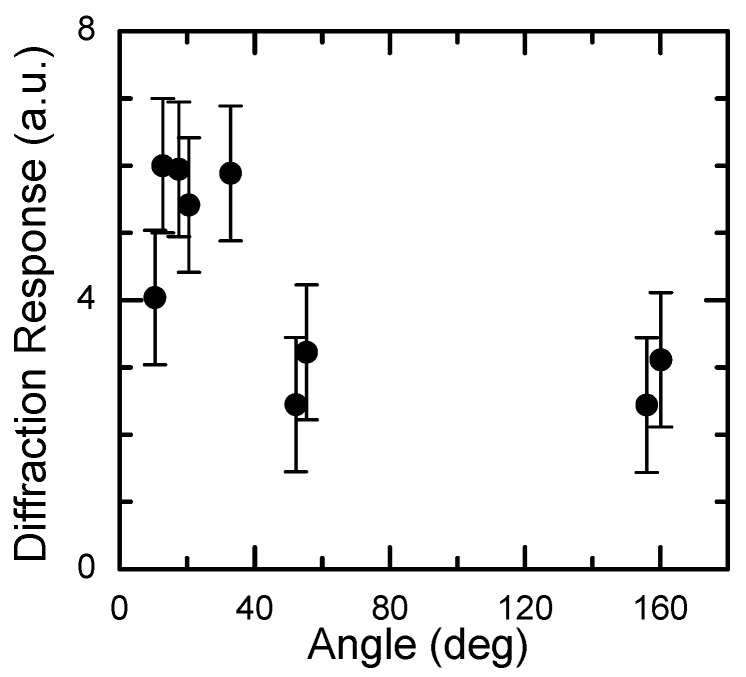
Dependency of diffraction efficiency of hologram in CdF_2_:In (*N*_In_ = 4 × 10^18^ cm^−3^) crystal on the convergence angle of recording beams.

The recording locality ensures high resolution of CdF_2_ crystals with bistable centers. Figure 20 shows the diffraction efficiency of holograms recorded by ruby laser in CdF_2_:In crystal, with convergence angles of recording beams in the ranges of 10–60° (transmitting holograms) and 150–160° (reflecting holograms). As follows from this figure, this angle does not practically influence the diffraction efficiency. For the maximal angle of 160°, the space frequency of the grating is 2800 mm^−1^ in air (4300 mm^−1^ inside the crystal). Reflecting holograms were recorded in this crystal by argon laser at angle convergence close to 180°. This shows that the space frequency of gratings in this crystal exceeds 5000 mm^−1^.

The sensitivity of CdF:Ga,Y crystal to grating recording, *S* ≅ 4 cm/J, is much higher than that of the typical photorefractive crystal LiNbO3:Fe (*S* = 0.02–0.07 cm/J) and comparable with that of Bi12TiO20 (*S* ≅ 10 cm/J); however, it is still lower than the sensitivity of Polaroid photopolymer (*S* ≅ 20 cm/J). This comparison is, however, not completely correct because at room temperature CdF_2_:Ga,Y crystal exhibits rather short storage time, whereas LiNbO_3_:Fe and Polaroid photopolymer ensure the recording of permanent holographic gratings. A more suitable characteristic of this crystal would be a nonlinear susceptibility* χ*^(3)^, that can be estimated in CdF_2_:Ga,Y as ∼− 1.4 × 10^−10^ m^2^/V^2^ [44].

**Figure 21:**
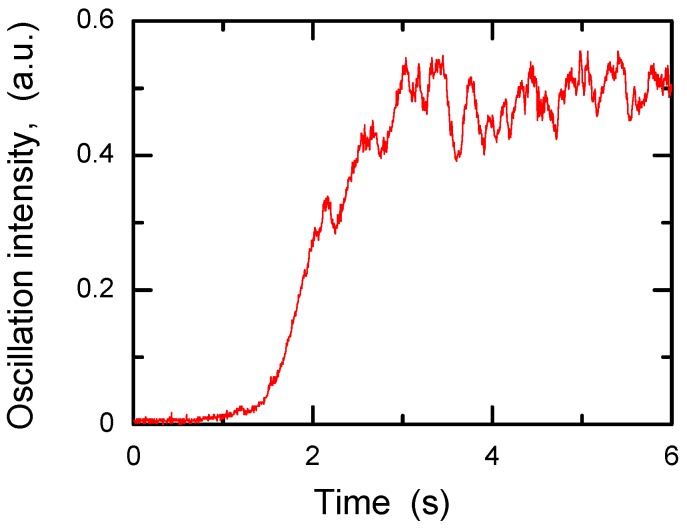
Temporal dynamics of coherent oscillation intensity in CdF_2_:Ga,Y (*N*_Ga_ = 5.7 × 10^17^ cm^−3^) crystal. Total intensity of counter-propagate pump beams ~ 200 mW/cm^2^ at equal intensities of these beams. Intensity fluctuations in the saturation area are due to poor mechanical stability of the optical table.

The high nonlinear susceptibility* χ*^(3)′^ of CdF_2_ crystals with bistable centers and high spatial frequency of recorded gratings makes these crystals a suitable object for the observation of backward-wave four-wave mixing, which is the underlying process for optical phase conjugation and also for various kinds of frequency-degenerate coherent optical oscillators. Phase conjugation and coherent optical oscillations in CdF_2_:Ga,Y crystal were reported in [49]. The interference of a relatively weak light beam coherent with two counter-propagate powerful (pump) beams formed the phase-conjugate mirror that, together with a conventional mirror, created the semi-linear cavity in which a coherent oscillator arose as a result of the interaction of signal and reversal weak waves. The temporal dynamics of oscillation intensity as a function of exposition time is shown in Figure 21. This dependency is characteristic for a coherent oscillator. The onset of oscillation proves unambiguously that the phase-conjugate reflectivity is higher than unity, *i.e.*, amplified reflection is reached.

Holograms can be recorded over the whole visible range of the spectrum, since for both crystals the UV-VIS band embraces all of this range. Their readouts with differing or the same wavelength determine two principle ways of using holographic elements based on CdF_2_ crystals with bistable centers. Both readout modes can be used in hologram recording by beams with the plane wavefront. Such recording allows formation of a static or dynamic (light-controlled) holographic mirror operating in both the visible and IR spectral ranges. The IR light-controlled holographic mirror based on CdF_2_:Ga crystal was used in a widely distributed Bragg reflector laser to set the lasing wavelength in the range of 1.260–1.285 µm [50].

Using the same wavelength for hologram recording and readout allows recording of dynamic information holograms operating in the visible range of the spectrum.

As demonstrated in [44], CdF_2_ crystals with bistable impurity centers can be used for dynamic recording of information patterns. In this paper the dynamic hologram of the 1951 USAF resolution test chart was successfully recorded and read-out in CdF_2_:Ga,Y crystal by continuous-wave Nd:YAG laser operating at *λ* = 532 nm at room temperature, which confirmed the high resolution of the medium.

In [51], holographic recording and readout of a binary information target (“chessboard-like” pattern) in CdF_2_:In crystal was performed at room temperature by frequency-doubled Nd:YAG-laser pulses with duration of 20 ns. According to the kinetics of the hologram decay (see Section 2.2), the decay time was about 0.1 ms in this case.

## 4. Holographic Optical Elements Based on Cdf_2_:Ga and Cdf_2_:in Crystals

### 4.1. Wavefront Correction

**Figure 22:**
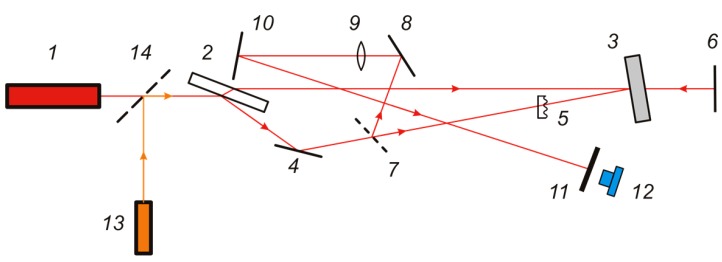
Optical scheme of the wavefront correction experiment. *1*: pulsed ruby laser; *2*: plane-parallel beam splitter plate; *3*: CdF_2_:In (*N*_In_ = 4 × 10^18^ cm^−3^) sample; *4*, *6*, *8*, *10*, *14*: plane mirrors; 5: model phase inhomogeneity; *7*: beam splitter; *9*: lens with 2 m focal length; *11*: dull screen; *12*: CCD-camera and line *13*: auxiliary adjusting He-Ne laser.

The dynamic phase conjugation ability of photochromic CdF_2_ crystals is demonstrated by an experiment on correction of model phase distortions with the use of a CdF_2_:In based wavefront-conjugating mirror [52]. The optical scheme of the experiment is shown in Figure 22. A ruby laser, *1*, is used as a source of pump and signal waves. To divide the output laser beam into signal and reference beams, a high-quality plane-parallel plate, *2*, was used. The angle of incidence of the beam on the plate and its orientation in space were chosen experimentally to provide the required ratio of beam powers. The cross sections of the concurrent pump beam, *E*_1_, and the signal beam, *E*_3_, were aligned in the CdF_2_:In crystal, *3*, which was in the form of a disk of 12 mm diameter and 1 mm thickness. The beam pass distances between the front face of the plate, *2*, and the crystal, *3*, for the waves *E*_1_ and *E*_3_ were the same and equal to 1540 mm. As a result, the beams were coherent in the crystal. The wave *E*_2_, formed due to reflection of the wave *E*_1_ from the plane mirror, *6*, was used as a readout beam.

The measuring scheme included a beam splitter, *7*, and a lens, *9*, with a focal length of 2 m, which focused the beam *E*_4_ reflected from a wavefront-conjugative mirror in the plane of the screen, *11*, after which a camera, *12*, was located. Figure 22 also shows the position of a model phase inhomogeneity, *5*, whose action was compensated for by a phase conjugation technique.

In the *Q*-switched mode of the source laser, the counter pump beam, *E*_1_, and signal beam, *E*_3_, had the energy of 40 mJ and their total power density in the crystal was 110 mJ/cm^2^. The transmittance of CdF_2_:In crystal at the ruby laser wavelength was 30%. Under these conditions, the reflectance of the wavefront-conjugating mirror in the absence of model phase distortion *κ* = 0.8%, while the diffraction efficiency of the reflection hologram recorded in the crystal *η* = 1.9%. The quality of the phase conjugated wave was found to be only insignificantly lower than that of the signal wave.

The time parameters of the wavefront-conjugating mirror were determined by analyzing the oscillograms of the laser and phase conjugated pulses. This analysis showed that the operating speed of the mirror under study was 10–20 ns.

In the case of wavefront-conjugating compensation for model phase distortions, the focal distribution of the phase conjugated wave was measured after the compensation for distortions. The similar distribution for the wave that passed twice through distortion without compensation was also recorded. In this case, the crystal, *3*, was replaced by a planar auxiliary mirror reflecting the signal wave exactly backward. For comparison, the phase conjugated wave in the absence of distortion at the same parameters was also recorded. This dataset allows determination of the quality of distortion compensation and the gain in the divergence as a result of the compensation.

**Figure 23:**
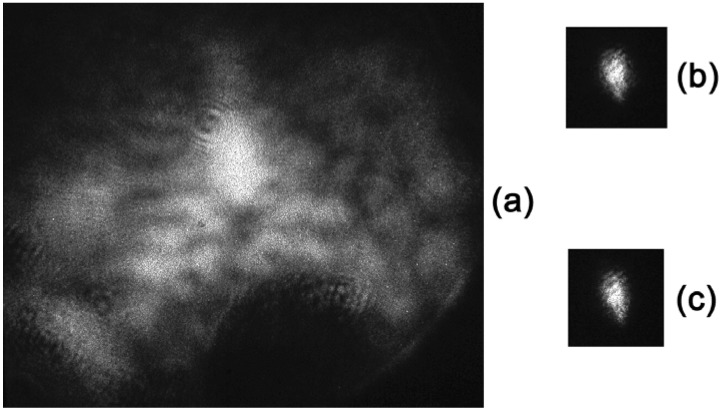
Results of compensation of a model phase distortion of the type of a spherical lens: (**а**) the focal distribution with a distortion of the type of a spherical lens (+1.25) and without phase conjugation; (**b**) the distribution with phase conjugation compensation of the lens, and (**c**) a phase conjugation wave without distortion. All images are given in the equal scale.

**Figure 24:**
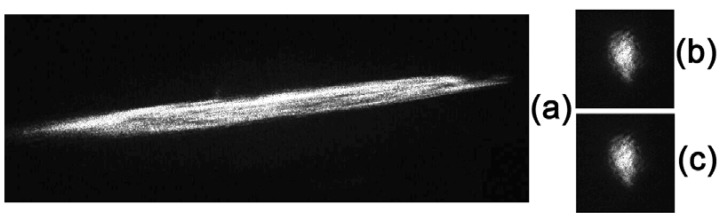
Results of compensation of a model phase distortion of the type of an astigmatic lens: (**а**) the focal distribution with a distortion of the type of an astigmatic lens (+1.25) and without phase conjugation; (**b**) the distribution with phase conjugation compensation of the lens and (**c**) a phase conjugation wave without distortion. All images are given in the equal scale.

Figure 23 and Figure 24 illustrate the distortion compensation for spherical and astigmatic lenses, respectively. The focal length of the lenses is 0.8 m. It can be seen that distortion is so large that, in the absence of compensation, the focal intensity distribution is distorted beyond recognition (Figure 23a and Figure 24a). With phase conjugated correction, the focal distribution is close to that in the absence of distortion; in this case, effective compensation of distortion occurs with a large gain in divergence. It should be noted that every picture shown in Figure 23 and Figure 24 was registered during a single 20 ns pulse of the ruby laser.

Volume reflecting holograms recorded in CdF_2_:Ga crystals were also used for correction of a model dynamic distortion of the wavefront [53].

### 4.2. Holographic Pattern Recognition

Another example of a CdF_2_-based holographic element application is a spatial-frequency filtration for dynamic optical image correlation. The volume nature of these filters ensures higher resolution compared to plane holographic filters due to high angular selectivity. It allows recognition of compared images with small angular difference. To obtain sufficiently large diffraction efficiency of the holographic filter, one needs to apply good-quality fringe pattern. To get such pattern in a volume sample with finite absorption (even in the isobestic gap) it is reasonable to record transmission holograms; in this case, intensities of interfering beams are equal though they change simultaneously along the sample thickness. For an optically isotropic medium such as CdF_2_ crystal, the use of parallel-polarized beams allows formation of high-contrast pattern at any beam convergence angle; in this case, polarization planes of the beams are perpendicular to the grating vector.

The dynamic matched spatial-frequency filter based on a CdF_2_:In crystal was used in a van der Lugt correlator scheme; the filter record and readout proceeded simultaneously by the same laser pulse but with 90°-shifted polarization [51].

**Figure 25:**
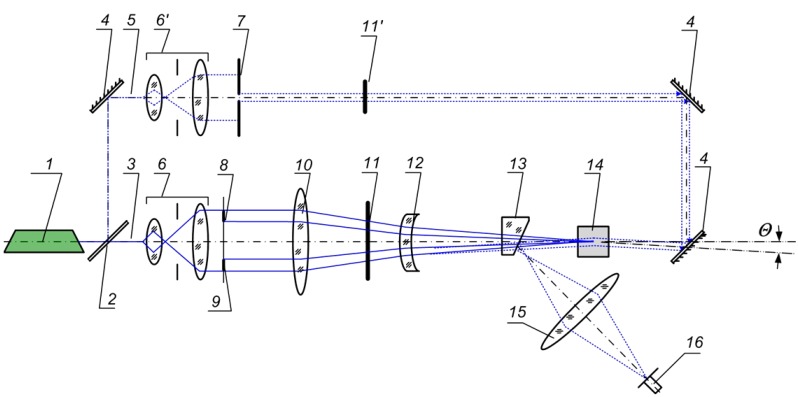
Optical scheme of the joint transform correlator based on a CdF_2_:Ga (*N*_Ga_ = 3 × 10^17^ cm^−3^) crystal: *1*: Ar laser; *2*: semitransparent mirror; *3*: recording beam; *4*: planar opaque mirror; *5*: reading beam; *6* and *6*′: collimators; *7*: diaphragm; *8* and *9*: information pattern (images *8 r*(*x*, *y*) and *9 s*(*x*, *y*)); *10* and *15*: Fourier transforming lens; *11* and *11*′: controlled gates; *12*: negative lens; *13*: optical wedge; *14* CdF_2_:Ga crystal; and *16*: CCD-camera.

The joint transform correlator (JTC) using a CdF_2_:Ga-based spatial-frequency filter [54] is shown in Figure 25. A 10 × 10 × 10 mm^3^ crystal with an antireflection coating on the working faces was used; the concentration of photochromic (optically active) centers was ~10^17^ cm^−3^. The power density of the recording/readout argon laser operating at 488 nm in the recording channel was ~100 mW/cm^2^, and the readout beam power was approximately 5% of the recording beam power. The hologram diffraction efficiency at room temperature was several percent, and the hologram decay time was several seconds.

In the recording channel of the JTC, the laser beam is expanded to the required diameter using a collimator, *6*, and the plane wavefront of this beam illuminates an information pattern with the images of reference (*r*(*x*, *y*), *8*) and analyzed (*s*(*x*, *y*), *9*) objects, located at the front focus of lens *10*. Lenses *10* and *12* perform Fourier transformation of the pattern and an interference pattern is recorded in the frequency plane of the JTC within a CdF_2_:Ga crystal, *14*; thus, a holographic spatial-frequency filter is formed in the crystal volume.

To recognize the reference image among analyzed ones, the plane wavefront, formed in the reading channel illuminates the crystal in the direction opposite to the recording beam from the *r(x*,* y*) image field. The reading beam reconstructs conjugated wavefronts of images coincident with the reference one. An optical wedge, *13*, directs the diffracted beam to the detection channel, in which lens *15* performs the second Fourier transformation and focuses the correlation signal at the correlator output plane, where it is detected by grayscale CCD-camera, *16*. The dynamic mode of the JTC operation is provided by computer-controlled mechanical gates, *11* and *11*′. These gates enable time separation of the recording and reading beams illuminating the crystal located in the frequency plane. This allows vanishing of scatter from the recording channel light in the detection channel. The gate operation time, determined by the hologram decay time, makes it possible to perform correlation at switching frequencies of the recording/reading channels in the range of 0.1–1 Hz.

The JTC optical scheme is based on a standard 4*f* correlator scheme; however, one should note its features: (1) introduction of an additional negative lens, *12*, increases the effective focal length of the Fourier transform and dimensions of the recording beam caustic; it makes possible to expose a larger crystal volume during hologram recording, thus preventing overexposure of the crystal and decreasing recording beam distortions; (2) the dynamic nature of the holographic medium enables changing of information patterns with compared objects at the correlator input plane in real time; one does not need to perform forced erasure of a previously written hologram or change the recording medium.

**Figure 26:**
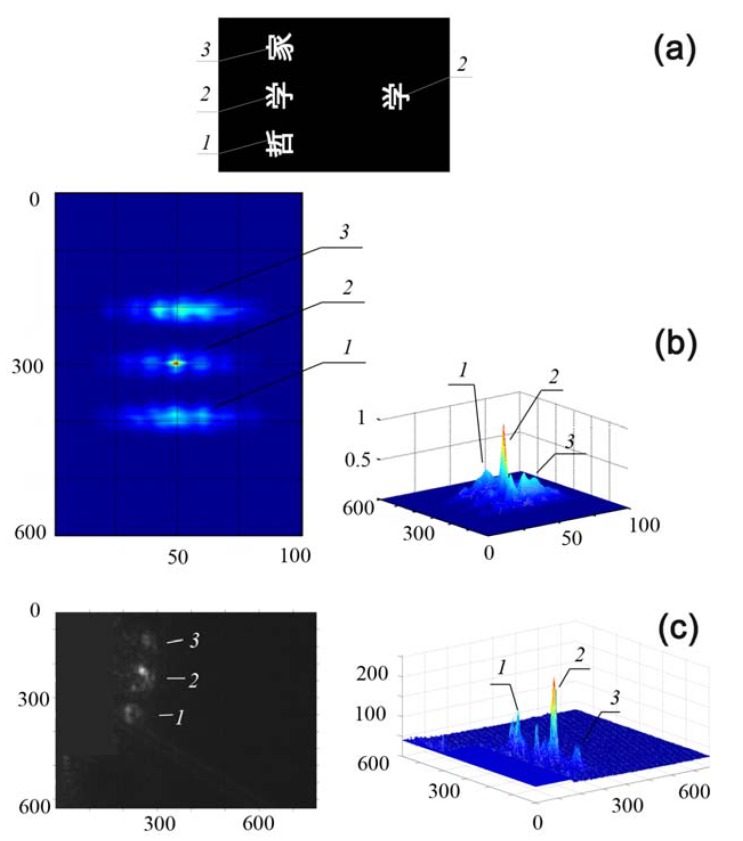
Recognition of hieroglyph *2* in a group of objects: (**a**) information pattern; (**b**) two-dimensional (left) and three-dimensional (right) images of calculated correlation field (signal coordinates are measured in pixels, signal intensities are measured in arbitrary units); (**c**) two-dimensional (left) and three-dimensional (right) experimental signal intensity distribution in the JTC output plane (signal coordinates are measured in pixels, signal intensities are measured in units of grayscale).

Binary and grayscale amplitude information patterns (transparencies) were used in the JTC. Both classes of transparencies were 24 × 36 mm^2^ in size (photographic frame format) and had a resolution of 25 pixels/mm. In Figure 26, the sample of selective object recognition, *i.e.*, recognition of a specified object in a group of objects presented for recognition, is shown. In this case, the reference object *r*(*x*) is a binary image of the Chinese hieroglyph 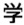 (*2*), located on the right-hand side of a transparency (Figure 26a) and the analyzed objects *s*(*x*) are three hieroglyphs placed on the left-hand side of the transparency (*1*, *2*, *3*).

According to the simulation data (Figure 26b), three peaks should be observed in the correlator output plane: one peak with the largest amplitude and the least half-width corresponds to the correlation signal of the reference object, *2*, while the two other peaks arise due to the cross-correlation of the reference with the other two hieroglyphs (*1* and *3*). The experimentally obtained signal intensity distributions in the correlator output plane (Figure 26c) really contain three peaks. As one can see, the amplitude of the correlation peak is indeed larger and its half-width is, accordingly, smaller than that of the cross-correlation peaks; the positions of the experimental peaks in the output plane correspond to the positions of the hieroglyphs compared. The shapes of the experimentally observed peaks differ from the calculated ones; this difference may be related to nonuniformity of laser illumination of the transparency field as well as aberrations of the optical scheme elements. However, one can easily perform threshold processing of the output JTC signals and thus detect the reference object among others presented for recognition.

**Figure 27:**
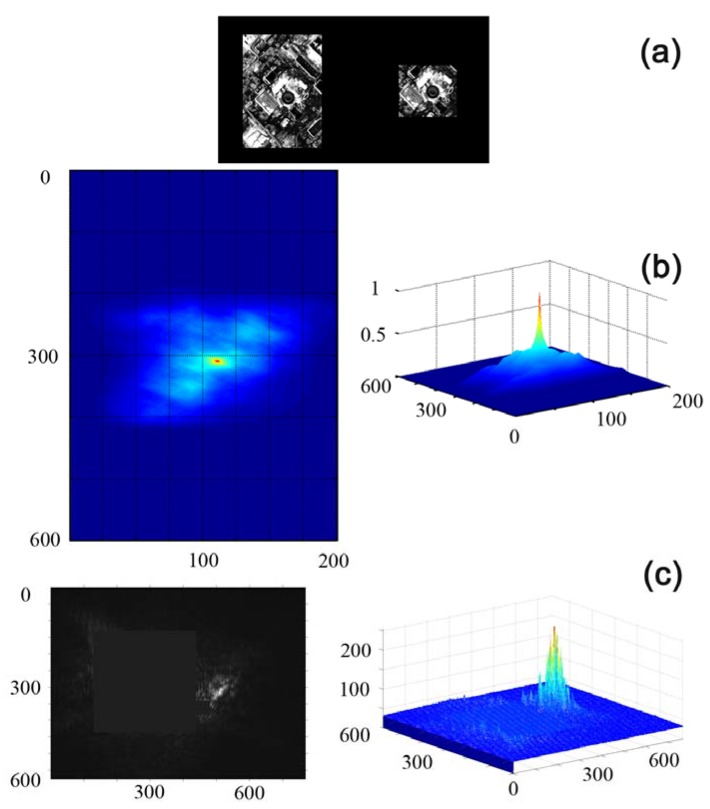
Correlation analysis of a satellite photograph of a commercial development region; the sequence order (**a**–**c**) and measuring units are as in Figure 26.

Another example of JTC performance is the recognition of an industrial object in the landscape environment or development elements in a satellite grayscale photograph, taken from the Google Map database (Figure 27).

The object under recognition is a round building with a rectangular extension. As one can see in Figure 27, the shape and the position of the experimentally observed correlation signal correspond sufficiently well to the calculated data.

The experimental JTC based on CdF_2_:Ga crystal demonstrates good ability for selective recognition when operating with objects of different complexity. It should be noted that the JTC operation rate can be increased by heating the CdF_2_:Ga crystal. CdF_2_ crystals possess higher resolution and operation speed as compared with widely used liquid crystals. To obtain high operating speed in combination with an acceptable hologram diffraction efficiency, it is not necessary to apply additional heating and/or external illumination or a high-voltage electric field to CdF_2_ crystal as for photorefractive crystals.

Bearing in mind the use of optical materials in correlators, one should note two modern trends of optical correlator buildup: the first involves the widespread use of liquid crystal light valves or spatial light modulators, which carry calculated spatial-frequency spectra of correlated patterns in the Fourier plane; the second is based on the use of a numerical correlation experiment instead of a physical one. The latter approach assumes that the correlation signal is calculated on the basis of known Fourier spectra of patterns and parameters of a virtual “optical scheme” of the correlator under study. In this case, one can easily implement additional spatial-frequency filters to the “scheme” and thus vary correlation signal characteristics [55]. However, such additional filters can increase the discrimination ability of traditional analogous correlators which use holographic media. For instance, one can make a hybrid wavelet-joint transform correlator in which additional wavelet-transform filters are employed, making the JTC less sensitive to scale and rotational non-invariances. Moreover, wavelet filtration enables to the increase of signal-to-noise ratio and correlation peak sharpness due to its edge enhancement nature which is useful for target recognition [56,57,58]. Thus, CdF_2_ crystals with bistable impurity centers can be considered as appropriate holographic media for fast hybrid wavelet-joint transform correlators.

## 5. Conclusions

The photochromy of CdF_2_ crystals with bistable impurity centers is based on the local modification of optical properties of the crystal that is stipulated by the reconstruction of the impurity center at the photoinduced change of its charge state. The potential barrier separating the ground (deep) and excited (shallow) states of the center determines the metastable nature of its excited state. The photoinduced deep-to-shallow center conversion underlies the hologram recording in these crystals. The hologram decay is tied to the thermally induced process of reverse (nonequilibrium shallow)-to-deep center conversion. These two processes have different natures and elapse in different time ranges.

The characteristic time of the photoinduced deep-to-shallow state conversion is determined by the lattice rearrangement around the free carrier and the impurity ion. The conversion process also includes the capture of the photoinduced free carrier by the trivalent impurity ion. The concentration of these ions is high and the capture occurs for a very short time interval as well as the above-mentioned rearrangement of the lattice. Hologram formation typically takes time of the order of nanoseconds at ambient temperature; this time weakly depends on the temperature.

The shallow-to-deep state conversion corresponds to the formation of one deep center from two shallow centers. This conversion includes the capture of thermally induced free carriers produced by the thermal ionization of a shallow center by another shallow center with its subsequent conversion into a deep center. The hologram decay that is determined by this process obeys bimolecular kinetics, degenerated into monomolecular kinetics at the latest stage of decay or at sufficiently high temperature. The deep center formation due to capture of the second electron by a shallow center occurs when the potential barrier is overcome. As a result, the time of hologram decay strongly depends on temperature. It varies in a wide limit from infinity (for low temperatures) down to times of the millisecond or nanosecond range, depending on the specific dopant ion.

Two bistable impurities, Ga and In, differ strongly in several respects (the barrier height, the deep state energy, the dependency of population of center states on the temperature). These distinctions determine the characteristics of phase holograms recorded in CdF_2_:Ga and CdF_2_:In crystals in the range of the overlapping tails of the UV-VIS and IR absorption bands (the isobestic gap), in particular, decay time of the hologram. Using the temperature as a managing parameter one can use holograms to follow up optical processes in the second-millisecond (CdF_2_:Ga) or second-nanosecond (CdF_2_:In) time ranges with diffraction efficiency from tenths down to units of percent. Such holograms can be recorded in several-millimeter-thick crystals.

The locality of the recording process ensures high resolution of the media that reaches at least 5000 mm^−1^.

The cubic symmetry of the crystals allows the use of polarization of radiation, which is especially useful for high frequencies since it creates the opportunity to divide hologram recording and readout processes. An unlimited number of record/readout cycles are possible for these media.

Holographic optical elements based on materials with volume holographic gratings are of great interest for optical information storage and processing. They can serve as spatial-frequency filters for a variety of purposes, such as encrypted optical memory systems, neural network simulation, holographic interferometry, image distortion corrections using phase-conjugation or backward-wave four-wave mixing, and optical correlation and convolution operations for pattern recognition. Some of these applications demand both static and dynamic holographic elements, whereas others need elements operating only in real time.

We maintain that real-time holographic media based on CdF_2_ crystals with bistable centers allow efficient operation within wide time scale and spectral ranges.
